# Impact of lymphadenectomy on short- and long-term complications in patients with endometrial cancer

**DOI:** 10.1007/s00404-022-06396-5

**Published:** 2022-01-17

**Authors:** Louisa Proppe, Ibrahim Alkatout, Ricarda Koch, Sascha Baum, Christos Kotanidis, Achim Rody, Lars C. Hanker, Georgios Gitas

**Affiliations:** 1 Department of Gynecology, University Hospital Schleswig-Holstein, Campus Luebeck, Ratzeburger Allee 160, 23562 Lübeck, Germany; 2 Department of Gynecology, University Hospital Schleswig-Holstein, Campus Kiel, Kiel, Germany; 3 Department of Gynecology, Vivantes Humboldt, Berlin, Germany

**Keywords:** Endometrial cancer, Lymphatic complications, Lymphadenectomy, Surgical complications

## Abstract

**Introduction:**

Early endometrial cancer is primarily treated surgically via hysterectomy, adenectomy and, depending on tumor stage and subtype, lymphadenectomy. Systematic lymph node dissection is known to cause surgical complications. The aim of the present study was to investigate morbidity and mortality rates associated with lymphadenectomy in patients with endometrial cancer who underwent surgery in a routine clinical setting.

**Methods:**

We collected data from 232 patients who were operated for endometrial carcinoma between 2006 and 2018 at the University of Lubeck, Germany. Surgical complications were viewed in relation to surgical risk factors. Additionally, a questionnaire concerning long-term lymphatic complications and survival was completed. Survival was compared between patients who underwent lymphadenectomy (group I) and those who did not (group II).

**Results:**

Patients in group I needed revision surgery significantly more often due to postoperative complications (such as lymphoceles) compared to those in group II (*p* = 0.01). The results indicate more serious complications in patients who underwent a systematic lymphadenectomy and in those with lymph node metastases. 15% of patients who underwent a systematic lymphadenectomy had lymph node metastases. Recurrences occurred in 12.5% of cases and were significantly more frequent in patients who had undergone a lymphadenectomy, even if the lymph nodes were negative (*p* = 0.02). A comparison of survival data during the follow-up period revealed no significant difference. The study highlighted the need for a better preoperative risk stratification and the avoidance of lymphadenectomy for surgical staging alone.

**Supplementary Information:**

The online version contains supplementary material available at 10.1007/s00404-022-06396-5.

## Introduction

The incidence of endometrial cancer increased by 2–5% in the US from 1998 to 2013 [1, 2]. Therefore, reducing complication rates after endometrial cancer surgery would be very desirable. Endometrial cancer, the most common gynecological carcinoma, is associated with approximately 11,300 new diagnoses per year in Germany [2, 3].

Especially due to the increasing incidence of the metabolic syndrome, which is one of the prime risk factors for developing endometrial carcinoma, one may expect an ongoing increase in the occurrence of this cancer during the next decades. Additionally, the prevalence of obesity is on the rise and obesity is known to exert a harmful effect on surgical outcomes.

Gentle treatment has become a part of many oncologic therapies, such as those for breast cancer. The frequency of systematic lymphadenectomy has been reduced. Patients with clinically negative lymph nodes did not have different recurrence-free or overall survival intervals during a 25-year follow-up, regardless of whether a systematic lymphadenectomy was performed [4]. In fact, a systematic axillary lymph node dissection was not needed in specific patients with positive lymph nodes [5]. Thus, adverse outcomes directly caused by lymph node dissection, such as lymphedema, were reduced from 15.3% after axillary lymph node dissection to 3.3% after sentinel node biopsy [4]. Ongoing research has been focused on reducing the extent of treatment in endometrial cancer, despite clear differences between the lymphatic drainage of the uterus and that of the mammary glands [6].

Notwithstanding the value of lymphadenectomy as a surgical staging procedure in endometrial cancer, its therapeutic effect remains controversial [7]. On the one hand, lymph node metastases are a common occurrence in endometrial cancer. On the other hand, systematic lymphadenectomy may cause intra- and postoperative complications, which might impair the patients’ quality of life postoperatively. According to Leitao et al., 40.9% of patients receiving a systematic lymphadenectomy for endometrial cancer experience long-term lymphatic complications such as lymphocele and lymphedema; these complications are significantly less common in patients without systematic lymph node dissection. Patients with postoperative lymphedema reported a significantly poorer quality of life compared to those without lymphedema [8]. Systematic lymphadenectomy is associated with relatively high complication rates compared to hysterectomy with adenectomy alone, which is performed in cases of low-risk endometrial carcinoma. The most common complications of systematic lymphadenectomy are intraoperative bleeding, injury to neighboring organs, postoperative lymph cysts (up to 34.5% of the cases), and lower-limb edema [9, 10]. Since more than a half of the patients undergoing a lymphadenectomy (LD) do not have lymph node metastases, the procedure may not improve survival [11–13]. The technique of sentinel node biopsy has been refined over the last few years [14–16]. The sentinel node technique has been validated for use in endometrial cancer, but was not incorporated in the standard of care during the period of the present study [17].

In stage I endometrial carcinoma, systematic lymphadenectomy improves neither recurrence-free survival nor overall survival [18]. Endometrial cancer may be treated by laparoscopy or laparotomy [19]. Open surgery is known to cause higher complication rates than laparoscopic procedures [20].

Hitherto, we still do not know whether lymphadenectomy performed in high-risk patients does improve survival or only serves as a useful staging procedure. While the discussion concerning its survival advantage is still in progress, long-term complication rates have been a neglected problem.

The aim of the present study was to obtain evidence about relevant surgical risk factors and postoperative morbidity. A further aim was to determine the impact of systematic lymphadenectomy on surgical and oncological outcomes.

## Patients and methods

**Fig. 1:**
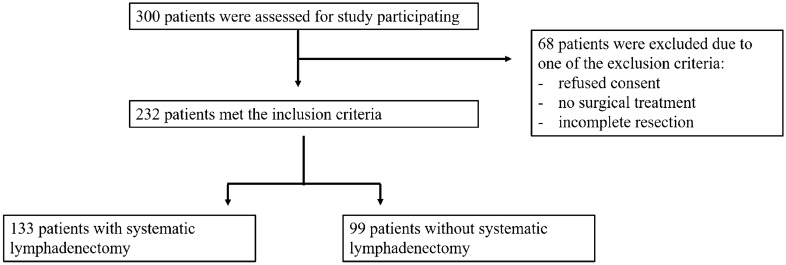
Consort diagram

A retrospective analysis was performed at the Department of Obstetrics and Gynecology, University of Lubeck, Germany. The medical files of all patients who underwent surgery for endometrial carcinoma from 2006 to 2018 were retrieved from the electronic information system of the hospital. Exclusion criteria were no surgical treatment, incomplete resection (R1), and the absence of patient consent. The study was performed in compliance with the Helsinki Declaration and approved by the ethics committee of the University of Luebeck (19-082A). Three hundred patients were recruited and 232 met the inclusion criteria. Patients were divided into two groups: group I consisted of those who had undergone a systematic lymphadenectomy (*n* = 133), and group II comprised patients with no systematic lymphadenectomy (*n* = 99) (Fig. 1).

A subgroup analysis was performed for comparison of clinical data. Patients who had undergone a systematic lymphadenectomy but had negative lymph nodes (*n* = 113) and patients without lymphadenectomy (*n* = 99) were compared. Additionally, surgical complications and risk factors such as BMI, diabetes mellitus, and nicotine abuse were analyzed.

Medical history, details of surgery, histology, tumor stage, and postoperative data were reviewed. The questionnaire was sent first to collect postoperative data. To assess treatment-related morbidity, the questionnaire addressed long-term surgical complications including lymphedema, symptomatic lymphocysts, pain related to surgery, and disease recurrence. Lymphocysts, wound healing disorders and lymphedema were defined as lymphatic complications. Patient data were analyzed in regard of independent risk factors such as nicotine abuse, previous surgery in the abdomen, the ECOG (Eastern Cooperative Oncology Group) performance status, parity, and diabetes mellitus. Surgical complications were analyzed separately for patients with a BMI higher than 30 kg/m^2^, and for those with positive versus negative lymph nodes after systematic lymph node dissection. Moreover, the overall (OS) and recurrence-free survival (progression-free survival, PFS) rates were evaluated.

The FIGO staging system was used to categorize disease. Pathologists make a distinction between type I, type II, and undifferentiated endometrial carcinoma. A systematic pelvic and para-aortic lymphadenectomy was performed in patients with intermediate- or high-risk endometrial cancer in accordance with the current German guidelines [17].

The choice between the laparoscopic and the open approach depended on the patient’s individual situation. The Clavien–Dindo classification was used to compare surgical complication rates [21]. Short-term postoperative complications were those that occurred up to 1 month after surgery. Grade I and II complications were considered mild, while Grade III–V complications were rated severe. The questionnaire was sent to the patients a second time to assess long-term complications. Patients without sufficient data records were excluded from the analysis.

Statistical analyses were performed using the free Python software, version 3.7, including the packages Pandas, Lifelines, SciPy, and NumPy [22].

To compare absolute and relative frequencies of clinical parameters, we used a variety of statistical tests depending on the scaling and distribution of variables. Fisher’s exact test was used for binary variables. The test yields the deviation of the result from the null hypothesis, and was proven valid even for small sample sizes. The chi-squared test was used for larger sample sizes with several categorical scaled variables. The chi-squared test demonstrates any significant differences between observed frequencies and expected frequencies in a contingency table. Finally, the Mann–Whitney *U* test was employed in some cases of ordinal scaled variables. It is a nonparametric test to compare differences between two probabilities. Survival data were analyzed using Kaplan–Meier-curves; confidence intervals indicate the degree of uncertainty.

## Results

Median age was similar in patients with (65.4 ± 11.5 years) and without lymphadenectomy (65.5 ± 13.5 years). The patients’ average BMI was 30.8 ± 8.6 kg/m^2^, which indicated an obese cohort. Overall, 42.9% of patients had a BMI higher than 30 kg/m^2^. Patients without lymphadenectomy were significantly more obese than those who had undergone systematic lymphadenectomy (*p* < 0.01). Previous surgeries have been mentioned as an independent risk factor for surgical complications. Approximately one half of the patients had undergone abdominal surgery prior to being diagnosed with endometrial cancer. The two groups did not differ significantly in regard of previous surgeries (*p* = 0.23). The majority of patients was postmenopausal (83%). On average, the patients had born 1.7 children; parity did not differ between groups. Tumor stages (according to the FIGO classification) did not differ significantly, except for those with FIGO III–IV disease. Of patients in the latter group, those who underwent lymphadenectomy were significantly more numerous than those who did not. Baseline characteristics are shown in Table 1, and the subgroup analysis in Table 2.

**Table 1:** Baseline characteristics and clinical data of patients with and without lymphadenectomy

	Patients with systematic lymphadenectomy (*n* = 133)	Patients without systematic lymphadenectomy (*n* = 99)	Total (*n* = 232)	*p *value
Age (average, years)	65.4 ± 11.5	65.5 ± 13.5	65.4 ± 12.3	n.s.
FIGO I–II	100 (43.1%)	93 (40.1%)	193 (83.2%)	n.s.
FIGO III–IV	33 (14.2%)	6 (2.6%)	39 (16.8%)	0.001
BMI (average, kg/m^2^)	29.4 ± 7.9	32.8 ± 9.3	30.8 ± 8.6	< 0.01
Revision surgery needed	31 (13.4%)	8 (3.4%)	39 (16.8%)	< 0.001
Average duration of surgery (minutes)	227.2 ± 97.4	132.8 ± 60.9	187.3 ± 96	< 0.001
Average duration of hospitalization	10.0 ± 7.4	6.0 ± 6.0	8.3 ± 7.2	< 0.001
Recurrence	22 (9.5%)	7 (3%)	29 (12.5%)	0.04
Lymphatic complication	19 (8.2%)	2 (0.9%)	21 (9%)	< 0.001
Surgical drain output (ml)	2439.3 ± 3212.0	247.6 ± 269.2	1502.9 ± 2664.6	< 0.001
Intraoperative blood loss (g/dl)	1.9 ± 1.3	1.3 ± 1.1	1.6 ± 1.3	0.002
Death during follow-up (data from 105/232 patients)	23/63 (36.5%)	8/41 (19.5%)	31/105 (29.5%)	n.s.

**Table 2:** Subgroup analysis of patients with lymphadenectomy but negative lymph nodes, and patients without lymphadenectomy

	Patients with negative lymph nodes (*n* = 113)	Patients without systematic lymphadenectomy (*n* = 99)	Total (*n* = 212)	*p* value
Age (average, years)	65.7 ± 11.3	65.5 ± 13.5	65.6 ± 12.3	n.s.
FIGO I–II	100 (47.2%)	93 (43.9%)	193 (91.0%)	n.s.
FIGO III–IV	13 (6.1%)	6 (2.8%)	19 (9.0%)	n.s.
BMI (average, kg/m^2^)	30.0 ± 7.9	32.8 ± 9.3	31.3 ± 8.7	< 0.001
Surgical complications* I–II	97 (45.5%)	89 (41.8%)	186 (87.3%)	n.s.
Surgical complications* III–IV°	16 (7.5%)	9 (4.2%)	25 (11.7%)	n.s.
Revision surgery	24 (11.3%)	8 (3.8%)	32 (15%)	0.01
Average duration of surgery (minutes)	221.9 ± 96.9	132.8 ± 60.9	180.9 ± 93.4	< 0.001
Average duration of hospitalization	9.9 ± 7.8	6.0 ± 6.0	8.1 ± 7.3	< 0.001
Recurrence	20 (9.4%)	7 (3.3%)	27 (12.7%)	0.02
Lymphatic complications	14 (6.6%)	2 (0.9%)	16 (7.5%)	0.004
Surgical drain output (ml)	2385.0 ± 3337.0	250.3 ± 269.2	1389.6 ± 2668.0	< 0.001
Intraoperative blood loss (g/dl)	1.8 ± 1.3	1.3 ± 1.1	1.6 ± 1.3	n.s.
Death during follow-up (data from 95/213 patients)	18/52 (18.9%)	8/43 (8.4%)	26/95 (27.4%)	n.s.

*According to the Clavien–Dindo classification

With regard to surgical technique, a conversion from laparoscopy to laparotomy was needed in 16 cases. Laparotomy was used in 82 patients and the laparoscopic approach in 150 patients. When a lymphadenectomy was performed, an average of 11.5 lymph nodes was removed. An endometrioid endometrial cancer was diagnosed in 219 patients, and a serous or clear cell histology in 13 patients. The mean duration of surgery was significantly longer when a lymphadenectomy was performed (*p* < 0.001); these patients also needed repeat surgery significantly more often because of postoperative complications such as lymphoceles (*p* < 0.001; Table 1). Performance status (ECOG) did not differ significantly between groups. Lymphatic complications were significantly more common in group I than in group II (*p* = 0.01). Furthermore, patients undergoing a lymphadenectomy had a significantly longer hospital stay than patients who did not (*p* < 0.001), and discharged significantly more fluid through the abdominal drain (*p* < 0.001). Patients receiving a systematic lymphadenectomy lost significantly more blood intraoperatively (*p* = 0.002, Table 1), and thus experienced significantly greater postoperative morbidity than those who had no lymphadenectomy.

Table 2 shows clinical characteristics of patients with a negative lymph node status after lymphadenectomy compared to patients without lymphadenectomy. Patients with negative lymph nodes after surgical staging had recurrent disease significantly more often than those without lymphadenectomy (*p* = 0.02, Table 2). Average complication rates did not differ significantly.

Surgical complications were categorized according to the Clavien–Dindo classification. No patient died due to surgery (Clavien–Dindo grade V). Despite the representative nature of the study sample, severe complications were rarely seen in the present investigation. The majority of patients had no or mild (grade I or II) complications. Surgical complications were analyzed with reference to various risk factors, as shown in Table 3.

**Table 3:** Surgical complications in relation to risk factors

Surgical risk factor	Surgical complications category I or II (*n* = 199)	Surgical complications category III (*n* = 23)	Surgical complications category IV (*n* = 9)
Diabetes mellitus (*n* = 42)	34 (17.1%)	5 (21.7%)	0
Nicotine abuse (*n* = 39)	35 (17.6%)	3 (13.0%)	1 (11.1%)
BMI ≥ 30 kg/m^2^ (*n* = 100)	89 (44.7%)	7 (30.4%)	3 (33.3%)
Systematic lymphadenectomy (*n* = 133)	110 (55.3%)	17 (73.9%)	6 (67.7%)
No lymphadenectomy (*n* = 99)	89 (44.7%)	9 (39.1)	0
Previously operated (no. of patients) (*n* = 113)	97 (48.7%)	12 (52.2%)	4 (44.4%)
Laparotomy (*n* = 82)	61 (30.6%)	14 (60.9%)	6 (66.7%)
Laparoscopy (*n* = 150)	128 (64.3%)	10 (43.5%)	5 (55.6%)

Given the rarity of severe complications, *p* values were not computed in the present analysis. A tendency towards more serious complications was noted in patients undergoing systematic lymphadenectomy and those with lymph node metastases. BMI or the presence of diabetes mellitus did not appear to have influenced the frequency of surgical complications (Table 3). Approximately 15% of patients who received a lymph node dissection were diagnosed with lymph node metastases; 35% of these experienced severe surgical complications while 17.3% of all patients who underwent a systematic lymphadenectomy experienced severe surgical complications.

Based on the questionnaire, the mean duration of follow-up was 59.5 months. During this time, 50% of patients were lost to follow-up. Thirty-one of these patients (13.4%) died during the follow-up period. The median OS and PFS of the entire group was 50 months and 36 months, respectively.

**Fig. 2:**
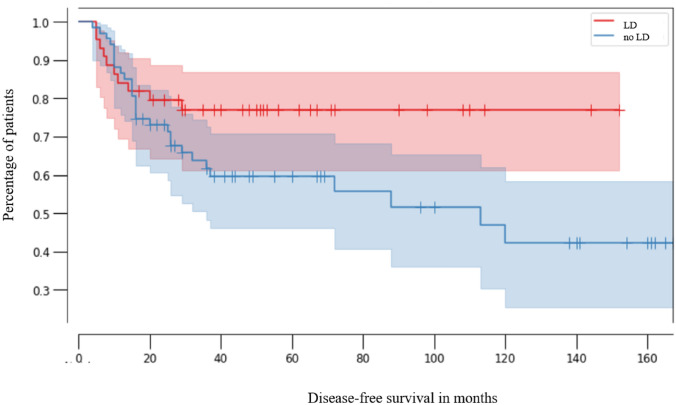
Kaplan–Meier analysis of progression-free survival in patients without lymphadenectomy (no LD, red line, *n* = 44, CI 0.99–0.61) and with lymphadenectomy (LD, blue line, *n* = 67, 95% CI 0.99–0.25, *p* = 0.07)

Patients with lymphadenectomy (LD) had a similar median PFS (29 months, 95% CI 0.99–0.25) as patients without lymphadenectomy (47 months, 95% CI 0.99–0.61; *p* = 0.07) (Fig. 2).

**Fig. 3:**
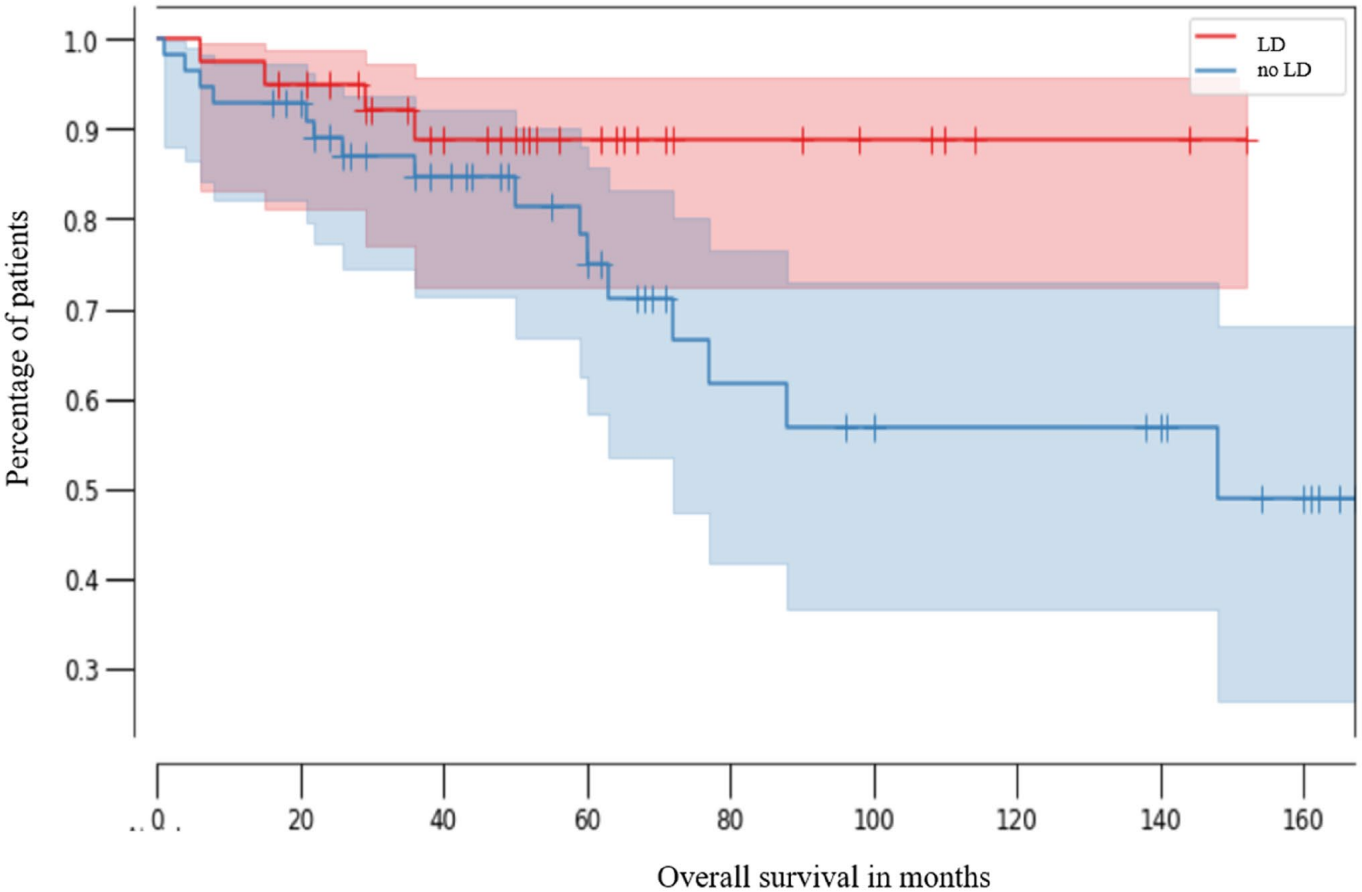
Kaplan–Meier analysis of overall survival in patients without lymphadenectomy (no LD, red line, *n* = 39, median 50 months, CI 0.99–0.72) and with lymphadenectomy (LD, blue line, *n* = 56, median 48.5 months, CI 0.99–0.26) (*p* = 0.07)

Overall survival did not differ significantly between patients with negative lymph nodes and those without lymph node staging. Median OS was 48.5 months in patients with lymphadenectomy (95% CI 0.99–0.26) versus 50 months in those without lymphadenectomy (95% CI 0.99–0.72, *p* = 0.07; Fig. 3). As shown by the 95% confidence intervals in Figs. 2 and 3, survival rates were associated with relatively high uncertainty. When comparing two groups, an overlap of confidence intervals indicates a statistically non-significant result. Consequently, the calculated differences between the two curves did not differ significantly.

## Discussion

We evaluated surgical complications, long-term morbidity (lymphatic complications and recurrences), and mortality in patients with endometrial cancer who underwent surgery with and without lymphadenectomy. According to the published literature, a systematic lymphadenectomy does not improve the prognosis of disease but may exert an effect on postoperative morbidity. We registered predominantly minor surgical complications and encountered no mortality as a result of surgery. Nevertheless, the high overall survival rate in patients with endometrial cancer (85–90% in the first 5 years) calls for treatment with minimized risks and complications [17, 23].

One of the foremost controversies in the treatment of endometrial cancer is lymphadenectomy. Two randomized phase-III trials yielded no survival benefit in patients with early endometrial carcinoma who underwent lymph node dissection [13, 18]. Nevertheless, systematic lymphadenectomy is an important tool for surgical staging.

The impact of lymph node dissection on surgical complication rates and long-term morbidity has been neglected for a long time. We performed a retrospective evaluation of potential risk factors for surgical complications, such as ECOG, blood loss, BMI, nicotine abuse, and diabetes mellitus, with regard to lymph node dissection in endometrial cancer.

The fact that lymphadenectomy causes significantly more surgical complications than a hysterectomy in combination with adenectomy for endometrial cancer was reaffirmed in the present study. Guidelines in Germany recommend the removal of at least 30 lymph nodes (pelvic and para-aortic) when performing a systematic lymphadenectomy. Although the average number of resected pelvic and para-aortic lymph nodes in our patients was relatively small (15.6 lymph nodes), we registered a significant number of severe long-term lymphatic complications. Patients who undergo a lymphadenectomy are at significantly higher risk of needing revision surgery than those without lymphadenectomy. In this study, the anatomical borders recommended for a pelvic lymphadenectomy include the iliac vessels as well as the external iliac lymph nodes and the interiliac and obturator lymph nodes. For a para-aortic lymphadenectomy, resection, and preparation up to the renal vessels is recommended. However, the total number of resected lymph nodes is relatively low. Aiming a reduction of lymphatic complications may have caused surgeon’s choice to spare some lymph nodes.

Furthermore, the impact of a learning curve when having established laparoscopical lymphadenectomy in endometrial cancer in the early years of the study might have influenced the average number of resected lymph nodes.

Scientifically, the impact of the extend of lymphadenectomy remains unclear. Recently, Xu et al. reported no influence of the total number of resected lymph nodes on patient’s survival, while other authors retrospectively pointed out a cutoff of > 10 nodes for a better survival [24, 25]. The present study confirmed that patients with lymphadenectomy develop lymphatic complications significantly more often than those without lymphadenectomy (*p* < 0.01). These complications impair quality of life, as shown in the GOG 244 trial [26]. The latter was one of the few prospective trials to evaluate lymphatic complications after lymphadenectomy in patients with gynecological cancers. Lymphatic complications reduce quality of life to a significant extent. In the GOG 244 trial, at baseline 96% of patients with lymph node dissection reported complications related to the lymphatic system. After 2 years, as many as 68% of patients still had some of those complications. Lymphatic complications aggravate the subjective experience of cancer distress. However, the authors of the GOG 244 trial registered no differences in sexual activity between patients with and without lymphadenectomy.

Severe complications were rare in our patients. However, all of those (100%) who received a lymphadenectomy experienced at least grade I surgical complications (self-limiting). As many as 73.9% of patients with a grade III surgical complication had undergone a lymphadenectomy. According to these data, lymphadenectomy is the most important risk factor for severe surgical complications. This is in line with the data reported by Togami et al., who identified lymphadenectomy itself as the main risk factor for lymphatic complications; other risk factors played a secondary role [27]. In our study, 35% of patients with lymph node metastases, but only 17.3% of those who had undergone a systematic lymphadenectomy experienced a severe surgical complication. These data suggest that patients with positive lymph nodes might experience more surgical complications than those with negative lymph nodes. Preoperative risk factors such as smoking or diabetes mellitus did not appear to have influenced the surgical outcome. In a prospective investigation, Swirska et al. also found that pre-existing diabetes mellitus did not affect postoperative outcomes in patients with gynecologic cancers [28].

The present study revealed that patients with systematic lymphadenectomy do not achieve better overall survival rates than those who do not undergo lymphadenectomy. One of the reasons may have been the small number of resected lymph nodes. Only 15% of our patients who underwent a systematic lymphadenectomy had positive lymph nodes (lymph node metastases). This is in line with other studies, in which a mere 15–20% of patients had positive lymph nodes after lymphadenectomy [29]. Muallem et al. reported positive lymph node metastases in 11.3–16.1% of patients with endometrial cancer in FIGO stages I or II but they did not report the total number of lymph nodes removed [30]. Our data concerning the prevalence of positive lymph nodes after lymphadenectomy in endometrial cancer are in line with those reported by Odagiri et al. in 2014 (15.8%), and Candido et al. in 2019 [31]. The latter authors registered no benefit in regard of disease-free or overall survival in patients with intermediate-risk endometrial carcinoma who had undergone a systematic lymphadenectomy with a median of 12 resected pelvic lymph nodes and 5 para-aortic ones. In contrary, Odagiri et al. reported a median number of 62.5 pelvic lymph nodes and 20 para-aortic lymph nodes. However, these differences of the total number of removed lymph nodes did not influence the survival rates.

Xu et al. evaluated, retrospectively, the effect of lymphadenectomy on survival rates in patients with (type I and type II) endometrial cancer. The authors aimed to determine the number of lymph nodes to be resected in order to achieve an overall survival benefit, and found no threshold for endometrioid endometrial carcinoma [24]. This is in line with our and the previously presented data, which indicate that the systematic lymphadenectomy does not improve overall survival rates. Further studies have also shown that lymph node dissection does neither improve overall survival nor progression-free survival in patients with endometrial cancer [13, 32, 33]. Benedetti et al. observed an overall survival rate of 85.9% in patients who underwent a lymphadenectomy versus 81.7% in patients without lymphadenectomy. In line with these data, we registered an overall survival rate of 87% in both groups. In contrast, Saotome et al. recently reported significantly better overall survival rates in patients who had undergone systematic lymph node dissection. However, the above mentioned study differed markedly from others in respect of group sizes (only 27% of all patients had no lymphadenectomy), age, and FIGO stages [34].

The average BMI of 30.8 kg/m^2^ in our patients reflects that obesity is one of the major risk factors for developing endometrial carcinoma, independent of whether lymph node dissection is performed. Patients who did not receive a systematic lymphadenectomy were significantly more obese than those who did (*p* < 0.01). Possibly, the risks of surgery prompted surgeons to refrain from performing a systematic lymphadenectomy in morbidly obese women. The results may have been influenced by the long period of patient enrollment (2006–2018). Urunsak et al. discuss the complications of lymphadenectomy in morbidly obese women. In contrast to our data, the majority of their patients who underwent lymphadenectomy were operated on via laparotomy. The risk of postoperative complications is known to be highest when this approach is used. In the future, robot-assisted surgery will probably permit surgeons to use the laparoscopic approach even in morbidly obese patients and thus reduce surgical complications [29, 35, 36]. Additionally, the sentinel node biopsy has recently become a standard procedure and is recommended by the latest ESGO guideline [37]. The sentinel technique, utilizing ICG mapping, allows an adequate and reliable surgical staging in patients with endometrial cancer, and reduces at the same time significantly postoperative complications [8, 38–41]. Hence, its use will increase within the next years. In our cohort, patients who underwent only a sentinel node biopsy and no following lymphadenectomy were excluded from the analysis. The limitations of the present study are its retrospective nature and the relatively small sample size; a half of the patients were lost in follow-up. However, the results serve as a contribution to the ongoing discussion about treatment-related morbidity and mortality in patients with endometrial cancer. The sentinel technique enables the surgeons to perform an adequate surgical staging without the necessity of a systematic lymphadenectomy and thus, reducing surgical complications.

We conclude that, in patients with early endometrioid endometrial cancer and clinically unsuspicious lymph nodes, a systematic lymph node dissection causes clinically relevant short- and long-term complications. Furthermore, a lymphadenectomy may have no impact on recurrence and overall survival rates. The data highlight the need for better preoperative risk stratification and surgical procedures such as sentinel node biopsy. The usefulness of radical lymphadenectomy may possibly be limited to surgical staging.

## Supplementary Information

Below is the link to the electronic supplementary material.

Supplementary file1 (DOCX 23 kb)

## Data Availability

Data are available at the corresponding author.
