# Supplementary material for: Impact of lymphadenectomy on short- and long-term complications in patients with endometrial cancer
Source: Arch Gynecol Obstet. 2022 Jan 17;306(3):811–9. doi: 10.1007/s00404-022-06396-5 (PMC9411244; doi:10.1007/s00404-022-06396-5)
Supplement: Supplementary file 1 — Supplementary file1 (DOCX 23 kb) [file 404_2022_6396_MOESM1_ESM.docx]

S1. Distribution of clinical FIGO stages in the study population. Percentages indicate relative mortality rates with reference to FIGO stage and lymphadenectomy.

|  | FIGO I | | FIGO II | | FIGO III | | FIGO IV | | *p*-value |
| --- | --- | --- | --- | --- | --- | --- | --- | --- | --- |
| No. of patients included in the study | 170 | | 22 | | 34 | | 5 | | - |
| No. of patients who died during follow-up | 19 (11.8 %) | | 4 (18.2 %) | | 7 (20.6 %) | | 1 (20 %) | | n.s. |
| Lymph-adenectomy | Yes | No | Yes | No | Yes | No | Yes | No |  |
|  | 82 | 88 | 18 | 5 | 30 | 4 | 3 | 2 | - |
| No. of patients who died during follow-up | 13 (15.9 %) | 6 (6.8 %) | 3 (16.7 %) | 1 (20 %) | 6 (20 %) | 1 (25 %) | 1 (33.3 %) | 0 | - |
